# Supplementary material for: Root morphological and physiological characteristics in maize seedlings adapted to low iron stress
Source: PLoS One. 2020 Sep 17;15(9):e0239075. doi: 10.1371/journal.pone.0239075 (PMC7498006; doi:10.1371/journal.pone.0239075)
Supplement: S1 Table — (DOCX) [file pone.0239075.s002.docx]

**Table S1 List of maize cultivars**

| Cultivar No. | Cultivar name | Breeding institution | Approved year |
| --- | --- | --- | --- |
| 1 | SAU1210 | Maize Research Institute of Sichuan Agricultural University | 2013 |
| 2 | Bisheng 2 | Dazhu Yimin Maize Research Institute | 2013 |
| 3 | Boyu 1 | Zizhong Ruibo Crop Seed Research Institute | 2005 |
| 4 | Chengdan 30 | Sichuan Academy of Agricultural Sciences | 2004 |
| 5 | Chuandan 189 | Maize Research Institute of Sichuan Agricultural University | 2011 |
| 6 | Chuandan 418 | Maize Research Institute of Sichuan Agricultural University | 2007 |
| 7 | Chuandan 455 | Maize Research Institute of Sichuan Agricultural University | 2013 |
| 8 | Deyu 18 | Beijing De Nong Seed Industry Co., Ltd. | 2004 |
| 9 | Denghai 605 | Shandong Denghai Seed Industry Co., Ltd. | 2010 |
| 10 | Difeng 998 | Sichuan Difeng Agricultural Technology Co., Ltd. | 2012 |
| 11 | Fangyu 1 | Dazhu Yimin Maize Research Institute | 2012 |
| 12 | Fude 2 | Sichuan Institute of Seed Engineering Technology | 2010 |
| 13 | Handan 999 | Sichuan Academy of Agricultural Sciences | 2012 |
| 14 | Huanongyu 8 | Beijing Huanong Weiye Seed Technology Co., Ltd. | 2010 |
| 15 | Huashi 9528 | Sichuan Huafeng Seed Industry Co., Ltd. | 2010 |
| 16 | Jiahe 158 | Weichang Manchu Mongolian Autonomous County Jiahe Seed Industry Co., Ltd. | 2012 |
| 17 | Kemao 918 | Nanchong Academy of Agricultural Sciences | 2012 |
| 18 | Miandan 118 | Mianyang Institute of Agricultural Sciences, Sichuan Province | 2010 |
| 19 | Quanyu 9 | Yunnan Longrui Seed Industry Co., Ltd. | 2011 |
| 20 | Qunce 99 | Dazhu Yimin Maize Research Institute | 2012 |
| 21 | Xianyu 508 | Tieling Pioneer Seed Research Ltd. | 2005 |
| 22 | Yifeng 311 | Sichuan Academy of Agricultural Sciences | 2010 |
| 23 | Changyu 19 | Shanxi Academy of Agricultural Sciences | 2009 |
| 24 | Zhengda 619 | Xiangfan Zhengda Agricultural Development Co., Ltd. | 2006 |
| 25 | Zhenghong 102 | Agricultural College of Sichuan Agricultural University | 2012 |
| 26 | Zhenghong 211 | Agricultural College of Sichuan Agricultural University | 2013 |
| 27 | Zhenghong 212 | Agricultural College of Sichuan Agricultural University | 2005 |
| 28 | Zhenghong 2 | Agricultural College of Sichuan Agricultural University | 2005 |
| 29 | Zhenghong 311 | Agricultural College of Sichuan Agricultural University | 2006 |
| 30 | Zhenghong 505 | Agricultural College of Sichuan Agricultural University | 2008 |
| 31 | Zhengtian 1 | Nanchong Academy of Agricultural Sciences | 2008 |
| 32 | Zhongdan 808 | Chinese Academy of Agricultural Sciences | 2006 |
