# Supplementary material for: Root morphological and physiological characteristics in maize seedlings adapted to low iron stress
Source: PLoS One. 2020 Sep 17;15(9):e0239075. doi: 10.1371/journal.pone.0239075 (PMC7498006; doi:10.1371/journal.pone.0239075)
Supplement: S2 Table — (DOCX) [file pone.0239075.s003.docx]

**Table S2 Differences in the low Fe tolerance coefficient of maize hybrids.**

| Cultivars | PH | SD | VL | LA | RDW | SDW | DWP | RSR | RCC | LAFC | FC | FA | RV | TRL | PRL | NRT | RSA | RD |
| --- | --- | --- | --- | --- | --- | --- | --- | --- | --- | --- | --- | --- | --- | --- | --- | --- | --- | --- |
| 1 | 0.96 | 0.88 | 0.89 | 0.93 | 0.77 | 0.76 | 0.76 | 1.02 | 0.97 | 1.20 | 0.79 | 0.60 | 0.67 | 0.47 | 0.67 | 0.54 | 0.55 | 1.22 |
| 2 | 0.75 | 0.93 | 0.81 | 0.49 | 0.78 | 0.48 | 0.54 | 1.63 | 0.37 | 0.69 | 0.67 | 0.36 | 0.91 | 0.31 | 0.68 | 0.19 | 0.52 | 1.78 |
| 3 | 0.85 | 1.02 | 1.00 | 0.85 | 0.53 | 0.62 | 0.59 | 0.95 | 0.98 | 0.88 | 0.64 | 0.36 | 0.59 | 0.43 | 0.67 | 0.39 | 0.51 | 1.19 |
| 4 | 0.84 | 0.90 | 0.94 | 0.62 | 0.80 | 0.59 | 0.62 | 1.33 | 0.68 | 0.65 | 0.47 | 0.29 | 1.03 | 0.58 | 0.69 | 0.38 | 0.76 | 1.33 |
| 5 | 0.64 | 0.77 | 0.80 | 0.40 | 0.72 | 0.39 | 0.45 | 1.85 | 0.24 | 1.19 | 0.63 | 0.28 | 0.70 | 0.51 | 0.47 | 0.52 | 0.60 | 1.19 |
| 6 | 0.83 | 0.83 | 0.83 | 0.78 | 0.70 | 0.51 | 0.55 | 1.35 | 0.53 | 0.98 | 0.33 | 0.18 | 0.79 | 0.50 | 0.71 | 0.41 | 0.63 | 1.26 |
| 7 | 0.66 | 0.83 | 0.78 | 0.53 | 0.64 | 0.43 | 0.46 | 1.49 | 0.28 | 0.78 | 0.96 | 0.44 | 0.59 | 0.37 | 0.40 | 0.34 | 0.47 | 1.26 |
| 8 | 0.97 | 0.90 | 0.90 | 0.80 | 0.62 | 0.70 | 0.68 | 0.88 | 0.99 | 0.81 | 0.42 | 0.29 | 0.66 | 0.62 | 0.94 | 0.59 | 0.64 | 1.04 |
| 9 | 0.97 | 0.86 | 0.83 | 0.83 | 0.71 | 0.78 | 0.76 | 0.91 | 0.90 | 1.30 | 0.42 | 0.32 | 0.74 | 0.59 | 0.88 | 0.60 | 0.66 | 1.13 |
| 10 | 0.99 | 0.91 | 0.89 | 0.97 | 0.71 | 0.71 | 0.71 | 1.01 | 0.90 | 1.36 | 0.34 | 0.24 | 0.97 | 0.84 | 1.37 | 0.66 | 0.89 | 1.11 |
| 11 | 0.95 | 0.86 | 1.00 | 0.74 | 0.68 | 0.72 | 0.71 | 0.94 | 0.69 | 1.56 | 0.33 | 0.24 | 0.63 | 0.87 | 0.93 | 0.66 | 0.74 | 0.85 |
| 12 | 0.69 | 0.85 | 1.07 | 0.54 | 0.57 | 0.53 | 0.54 | 1.08 | 0.47 | 0.89 | 0.43 | 0.23 | 0.64 | 0.61 | 0.68 | 0.53 | 0.62 | 1.03 |
| 13 | 0.97 | 0.74 | 0.83 | 0.89 | 0.86 | 0.78 | 0.80 | 1.10 | 0.99 | 1.02 | 0.50 | 0.40 | 0.82 | 0.76 | 0.80 | 0.60 | 0.79 | 1.04 |
| 14 | 0.98 | 0.93 | 1.00 | 1.09 | 0.82 | 0.87 | 0.86 | 0.94 | 0.98 | 1.03 | 0.42 | 0.36 | 0.98 | 0.68 | 0.84 | 0.67 | 0.80 | 1.25 |
| 15 | 0.95 | 0.81 | 1.07 | 0.85 | 0.71 | 0.72 | 0.72 | 0.98 | 0.99 | 1.29 | 0.31 | 0.22 | 0.65 | 0.64 | 0.81 | 0.63 | 0.64 | 1.00 |
| 16 | 0.89 | 0.81 | 1.00 | 0.66 | 0.79 | 0.75 | 0.76 | 1.04 | 0.81 | 1.43 | 0.76 | 0.57 | 0.84 | 0.77 | 0.72 | 0.61 | 0.80 | 1.04 |
| 17 | 0.93 | 1.09 | 0.94 | 0.97 | 0.80 | 0.73 | 0.75 | 1.09 | 0.74 | 0.91 | 0.41 | 0.29 | 0.91 | 0.75 | 0.83 | 0.67 | 0.83 | 1.10 |
| 18 | 0.77 | 0.91 | 0.94 | 0.68 | 1.13 | 0.55 | 0.65 | 2.08 | 0.62 | 0.89 | 0.78 | 0.51 | 1.01 | 0.66 | 0.82 | 0.54 | 0.82 | 1.24 |
| 19 | 1.00 | 0.90 | 0.83 | 1.03 | 0.87 | 0.91 | 0.90 | 0.96 | 0.93 | 0.65 | 0.53 | 0.48 | 0.78 | 0.83 | 1.08 | 0.78 | 0.80 | 0.97 |
| 20 | 0.82 | 0.83 | 0.83 | 0.64 | 0.64 | 0.57 | 0.58 | 1.12 | 0.63 | 0.54 | 0.69 | 0.40 | 0.77 | 0.46 | 0.71 | 0.49 | 0.59 | 1.30 |
| 21 | 0.89 | 0.88 | 0.89 | 0.72 | 0.92 | 0.64 | 0.69 | 1.42 | 0.53 | 1.71 | 0.53 | 0.37 | 0.96 | 1.09 | 1.00 | 1.03 | 1.02 | 0.94 |
| 22 | 0.88 | 0.79 | 0.83 | 0.77 | 0.64 | 0.74 | 0.72 | 0.88 | 0.86 | 0.49 | 0.49 | 0.35 | 0.61 | 0.56 | 0.89 | 0.65 | 0.58 | 1.06 |
| 23 | 0.91 | 0.93 | 0.89 | 0.84 | 0.73 | 0.63 | 0.65 | 1.16 | 0.55 | 0.77 | 0.38 | 0.25 | 0.91 | 0.53 | 0.82 | 0.36 | 0.69 | 1.30 |
| 24 | 1.06 | 0.99 | 0.90 | 0.81 | 0.70 | 0.76 | 0.74 | 0.92 | 0.99 | 0.92 | 0.40 | 0.29 | 0.77 | 0.70 | 0.99 | 1.12 | 0.73 | 1.04 |
| 25 | 0.64 | 0.86 | 0.89 | 0.47 | 0.61 | 0.54 | 0.55 | 1.15 | 0.35 | 1.02 | 0.29 | 0.16 | 0.56 | 0.51 | 0.61 | 0.37 | 0.54 | 1.04 |
| 26 | 0.82 | 0.78 | 0.83 | 0.65 | 0.56 | 0.47 | 0.48 | 1.19 | 0.31 | 1.16 | 0.53 | 0.25 | 0.60 | 0.69 | 0.59 | 0.60 | 0.64 | 0.93 |
| 27 | 0.85 | 0.93 | 0.94 | 0.84 | 0.71 | 0.72 | 0.71 | 0.99 | 0.97 | 0.88 | 0.31 | 0.15 | 0.36 | 0.44 | 0.56 | 0.26 | 0.40 | 0.91 |
| 28 | 1.02 | 0.91 | 1.00 | 0.84 | 0.78 | 0.83 | 0.82 | 0.94 | 0.98 | 0.72 | 0.33 | 0.27 | 0.75 | 0.76 | 1.18 | 0.68 | 0.75 | 1.01 |
| 29 | 0.53 | 0.72 | 0.77 | 0.33 | 0.53 | 0.30 | 0.34 | 1.76 | 0.32 | 0.76 | 0.61 | 0.21 | 0.48 | 0.37 | 0.43 | 0.48 | 0.42 | 1.13 |
| 30 | 0.93 | 1.09 | 1.07 | 0.85 | 0.60 | 0.69 | 0.67 | 0.87 | 0.51 | 1.20 | 0.25 | 0.16 | 0.72 | 0.78 | 0.80 | 0.72 | 0.75 | 0.95 |
| 31 | 0.99 | 1.00 | 0.96 | 1.01 | 0.89 | 1.03 | 0.99 | 0.87 | 0.86 | 0.93 | 0.39 | 0.38 | 0.97 | 0.84 | 0.80 | 0.97 | 0.91 | 1.07 |
| 32 | 0.84 | 0.99 | 1.00 | 0.83 | 0.91 | 0.86 | 0.87 | 1.05 | 0.87 | 1.38 | 0.48 | 0.39 | 0.94 | 0.69 | 0.75 | 0.94 | 0.80 | 1.14 |
| Average | **0.87** | **0.89** | **0.91** | **0.76** | **0.73** | **0.67** | **0.68** | **1.16** | **0.71** | **1.03** | **0.49** | **0.32** | **0.76** | **0.63** | **0.78** | **0.59** | **0.68** | **1.12** |

Note: PH: plant height; SD: stem diameter; VL: visible leaf; LA: leaf area; RDW: root dry weight; SDW: shoot dry weight; DWP: dry weight per plant; RSR: root-to-shoot ratio; RCC: relative chlorophyll content; LAFC: leaf active Fe content; FC: Fe content; FA: Fe accumulation; RV: root volume; TRL: total root length; PRL: primary root length; NRT: number of root tips; RSA: root surface are; RD: root diameter.
