# Supplementary material for: Root morphological and physiological characteristics in maize seedlings adapted to low iron stress
Source: PLoS One. 2020 Sep 17;15(9):e0239075. doi: 10.1371/journal.pone.0239075 (PMC7498006; doi:10.1371/journal.pone.0239075)
Supplement: Attachment — Submitted filename: Response to Reviewers.docx [file pone.0239075.s005.docx]

Dear Editors and Reviewers:

Thank you for your letter and for the reviewers’ comments concerning our manuscript entitled “Morphological and physiological characteristics of maize seedling adapted to low iron stress” (PONE-D-20-12291). Those comments are all valuable and very helpful for revising and improving our manuscript, as well as the important guiding significance to our researches. We have studied comments carefully and have made correction which we hope meet with approval. Revised portion are marked in red in the paper. The main corrections in the paper and the responds to the reviewer’s comments are as following:

**Responds to the reviewer’s comments:**

**Reviewer: 1**

Language: The present language quality is not good enough and needs to be improved. The manuscript would benefit from a thorough proofread by a native English speaker to improve grammar.

**Response to comment:** Our revised manuscript has been polished and revised by senior editor of Editage.

Abstract: I would prefer a results section with fewer details and numbers. The most important findings can be addressed, but I would not include all findings. The abstract is not well balanced.

**Response to comment:** We have revised the abstract to present some details and numbers.

Introduction: It is a good introduction. It contextualizes and gives important background knowledge.

**Response to comment:** There is no need to answer.

Materials and methods: it is good but the authors need to give more details about their experiments such as the number of replications, experimental design, and statistical analysis that they used in their experiments.

**Response to comment:** We added the number of replications, experimental design, and statistical analysis that they used in their experiments in the revised manuscript.

Results: Overall it is a good result and clear.

**Response to comment:** There is no need to answer.

Discussion: Overall it is a good discussion; I think the discussion would be even better if the authors use more appropriate references to have a good discussion.

**Response to comment:** We used some more appropriate references to have a good discussion in the revised manuscript.

Conclusion: the conclusion is too long; the authors can be reduced without losing the key.

**Response to comment:** We reduced the conclusion without losing the key.

References: some of the references are old [17] and [18]. All the references should be presented according to journal guidelines.

**Response to comment:** We replaced the old [17] and [18] with new references, and presented all the references according to journal guidelines.

**Reviewer: 2**

Abstract: Clearly add the treatments’ description. Levels of Fe supply? If possible, add the numeric description of some key parameters. Conclude the abstract section (Key findings in a single sentence)

**Response to comment:** We have revised the abstract, added Fe supply levels and numeric description of some key parameters.

Better to change the title, reporting key findings of your study.

**Response to comment:** We changed the title to focused on root morphological and physiological characteristics.

Introduction: Introduction section contains various unnecessary statements. Remain focused on the topic. Begin with the broadest scope and get progressively narrower.

Add a clear-cut research hypothesis.

Whether the Fe deficiency is a problem in southwest China. Add such information in introduction. I think, its not a problem in soils with pH<7.

Objectives should be specific and clear. Morphological and physiological parameters were only investigated for two cultivars (not for all).

**Response to comment:** We have revised the introduction section and added a clear-cut research hypothesis.

Sichuan Province is a big province of maize planting and consumption in China. More than 1/3 of dry land in the region is calcareous purple soil, and the content of available iron in soil is low, which seriously restricts the improvement of maize yield.

We have added this information in introduction.

Of course, iron deficiency is serious in calcareous soil (pH>7).

We have added the objectives. In this study, we selected two cultivars with the greatest difference in adaptability to low iron stress to study morphological and physiological parameters, mainly considering the workload and timeliness of the experiment. Of course, in the follow-up study, we are also considering the differences of all cultivars to present more systematic research results.

Methods:

It is necessary to clearly substantiate the use of two cultivars (Exp 2), since there are no explanations in the text of the article. It would nice if authors shortly indicate some major traits of chosen maize cultivars. Why these cultivars were selected to experiment? Based of which parameters?

L113: The seeds were then germinated (How many?) Similarly, how many seedlings were transferred to pots.

Add the details of software used.

If possible, add the brief methodology for the measurement of different parameters. How about sampling method/time/amount?

Clearly specify the details on statistical analysis separately for exp 1 and 2. It can not be same.

Information regarding experimental design is missing. Be consistent regarding the use of abbreviations…

**Response to comment:** The two cultivars are the biggest difference in iron efficiency through cluster analysis and principal component analysis based on the data in the first Experiment. We have added shortly indicate some major traits of chosen maize cultivars in the revised manuscript.

We added the number of seedlings transferred to pots in the revised manuscript.

We added the details of software used, and sampling method/time/amount in the revised manuscript.

We clearly specified the details on statistical analysis separately for exp 1 and 2 in the revised manuscript.

We added the experimental design information in the revised manuscript.

Results. Despite the large amount of results obtained by the authors, they look like a set of data that are not interconnected.

**Response to comment:** We reorganized the results section to make them more interconnected in the revised manuscript.

Language needs substantial improvement. There are several grammatical and typo mistakes. Many statements are confusing/unclear. e.g. L203: the decreases in plant height and stem diameter (compared with what?)

**Response to comment:** Our revised manuscript has been polished and revised by senior editor of Editage, and rewrite the confusing part in the revised manuscript.

Why the authors only measured only CAT and POD, if the data regarding other oxidative stress indicators (e.g ROS) is not present.

**Response to comment:** There are many oxidative stress indicators of plant (e.g. CAT, POD, SOD, ROS), we selected CAT and POD to reflect the differences between the two maize cultivars in the study, so not all the oxidative stress indicators were measured.

Discussion should be merely based on the observed findings Answer the question posed in introduction, and correlate your finding with the existing knowledge. Avoid the repetition. i.e description of results. Check whether the format of all references is according to the journal format.

**Response to comment:** We revised the discussion and formatted of all references according to the journal format.

Conclusion: Just report the key findings, it should not be detailed summary.

**Response to comment:** We reduced the conclusion.
