# Supplementary material for: Root morphological and physiological characteristics in maize seedlings adapted to low iron stress
Source: PLoS One. 2020 Sep 17;15(9):e0239075. doi: 10.1371/journal.pone.0239075 (PMC7498006; doi:10.1371/journal.pone.0239075)
Supplement: Attachment — Submitted filename: Response to Reviewers.docx [file pone.0239075.s007.docx]

Dear Editors and Reviewers:

Thank you for your letter and for the reviewers’ comments concerning our manuscript entitled “Root morphological and physiological characteristics of maize seedlings adapted to low iron stress” (PONE-D-20-12291R1). Those comments are all valuable and very helpful for revising and improving our manuscript, as well as the important guiding significance to our researches. We have studied comments carefully and have made correction which we hope meet with approval. Revised portion are marked in red in the paper. The main corrections in the paper and the responds to the reviewer’s comments are as following:

**Responds to the reviewer’s comments:**

**Reviewer: 1**

English must be enhanced

Even though suggestions in his respect were provided by former reviewers, they are not respected, and the manuscript was not edited as a result. The authors must take into account the above-mentioned suggestions and make the suitable corrections.

**Response to comment:** Although in the first revision, we have already revised the English of the full manuscript through *Editage*, which has not achieved good results, we are deeply sorry. After checking the reviewers' comments, we applied for *Editage* to revise the full manuscript English of the article, hoping to meet the requirements of publication.

**Reviewer: 2**

Be consistent regarding abbreviations. All the abbreviations should be well defined at first mentioned place. Line36, CAT and POD are unexplained.

**Response to comment:** We have revised it in the revised manuscript.

Keywords are not sufficient, and do not cover the main theme of ms.

**Response to comment:** We changed the keywords “Iron, Chlorophyll, mugineic acid, Zea mays” to “Iron, root morphology, physiological characteristics, *Zea mays* L.”.

Add brief methodology for the measurements of different morphological and physiological attributes.

**Response to comment:** In the part of “Sampling and measurements”, we have supplemented the brief methodology for the measurements of different morphological and physiological attributes.

In statistical analysis, probability level for mean comparison test?

**Response to comment:** Means were compared using least significant difference (LSD) test at the 0.05 level to determine the diﬀerences between the means of each treatment. It has been added in the revised manuscript.

In discussion: Discuss the activities of antioxidant enzymes in relation to Fe supply.

**Response to comment:** We have supplemented the discussion in the revised manuscript.

In Figure 6 captions: define 1 U for CAT and POD

**Response to comment:** We have defined 1 U for CAT and POD in the revised manuscript.
